# HIV in Indian prisons: Risk behaviour, prevalence, prevention & treatment

**Published:** 2010-12

**Authors:** Kate Dolan, Sarah Larney

**Affiliations:** *Program of International Research & Training, National Drug & Alcohol Research Centre, University of New South Wales, Sydney, Australia*

**Keywords:** Drug dependency, harm reduction, HIV, India, prisons, risk behaviour

## Abstract

**Background & Objectives:**

HIV is a major health challenge for prison authorities. HIV in prisons has implications for HIV in the general community. The aim of this paper was to gather information on HIV risk, prevalence, prevention and treatment in prisons in India.

**Methods:**

Relevant published and unpublished reports and information were sought in order to provide a coherent picture of the current situation relating to HIV prevention, treatment and care in prisons in India. Information covered prison management and population statistics, general conditions in prisons, provision of general medical care and the HIV situation in prison.

**Results:**

No data on drug injection in prison were identified. Sex between men was reported to be common in some Indian prisons. A national study found that 1.7 per cent of inmates were HIV positive. Some prisons provided HIV education. Condom provision was considered illegal. A few prisoners received drug treatment for drug use, HIV infection or co-infection with sexually transmitted infections (STIs).

**Interpretation & conclusions:**

HIV prevalence in prisons in India was higher than that in the general community. Regular monitoring of information on HIV risk behaviours and prevalence in Indian prisons is strongly recommended. Evidence based treatment for drug injectors and nation-wide provision of HIV prevention strategies are urgently required. Voluntary counselling, testing and treatment for HIV and STIs should be provided.

In 2008, around 5 million people in Asia were living with HIV. India accounts for about half of these infections^1^. Sentinel surveillance conducted by the National AIDS Control Organization (NACO) shows that in the general population HIV prevalence is low (0.25-0.43%), but among high-risk groups, HIV prevalence is much more. In at least five States, HIV prevalence among injecting drug users (IDU) is greater than 10 per cent, with a high of 24 per cent of IDUs are HIV positive in Maharashtra. Prevalence is also elevated among female sex workers and men who have sex with men^2^.

Globally, progress has been made in implementing HIV programmes in the community^2^; however, HIV prevention, care and treatment have largely been neglected in prisons^3^. HIV is a major health challenge for prison authorities^4^,^5^ because substance use disorders^6^ and injecting drug use^7^ are common among incarcerated populations. Subsequently, HIV, viral hepatitis and tuberculosis are more prevalent in prison populations than in the general population^8^.

There are an estimated 165,000 IDU in India^9^, and it is common for people who use illicit drugs to experience periods in custody^10^. However, there are very limited data on the prevalence of drug use or other HIV risk behaviours among Indian prisoners. In a study conducted in 1997-2000, around 8 per cent of individuals admitted to Tihar Jail in Delhi were known to be drug users^11^, while in a more recent study of 466 inmates in Delhi, Mumbai and Punjab, 63 per cent reported ever using illicit drugs^12^. Despite this uncertainty, it is highly likely that, as in most countries around the world^7^, people at high risk of HIV infection are over-represented in Indian prisons.

Prisons do not exist in isolation from the community. The majority of prisoners return to the cities and towns they came from. Resumption of risk behaviours such as unprotected sex^13^ and drug abuse^14^,^15^ shortly after release from prison is common. This study was carried out to collect information on HIV risk, prevalence, prevention and treatment programme in prisons in India.

## Material & Methods

Relevant published material was searched from 1993 to 2010 and unpublished information from key experts relating to HIV in prisons in India was obtained by request. Specific information included three main areas: (*i*) Rates of imprisonment; (*ii*) HIV testing, prevalence and risk behaviours; and (*iii*) HIV prevention, care and treatment.

The online databases Web of Science, PubMed and Scopus were searched using the search string “(HIV or AIDS or HIV/AIDS) and prison or jail or gaol or correctional and India”. Grey literature was accessed by searching the following websites: National Crime Records Bureau *http://ncrb.nic.in/*; The International Centre for Prison Studies *http://www.kcl.ac.uk/schools/law/research/icps*; International AIDS Society *http://www.iasociety.org/*; UNAIDS *http://www.unaids.org/en/*; UNODC Regional Centre for South Asia *http://www.unodc.org/india/index.html*; and WHO Regional Office for South-East Asia *http://www.searo.who.int/*. Only English-language documents were reviewed.

Where gaps in the data remained, key experts were contacted via e-mail or telephone and requests for specific information were made. Key experts included international agencies, non government organizations (NGOs) working in prisons and/or the community in target countries and officials from relevant government ministries and prison administrative bodies.

## Results & Discussion

*Prison population*: The latest figures available on Indian prison population were 376,396 adult prisoners for 2007^16^, making for an imprisonment rate of 32 per 100,000 of national population^17^.

Around two-thirds of inmates were pre-trial detainees^17^. A high proportion of pre-trial detainees means a very large inmate turnover. For example, in Tihar Jail, New Delhi, about 12,000 inmates were held while about 60,000 prisoners were admitted and a similar number were discharged annually^17^. Indian prisons were overcrowded. Across all correctional facilities, the average occupancy level was 136 per cent^16^. The three areas with the largest overcrowding were Uttar Pradesh (201%) followed by Chhattisgarh (193%) and Delhi (185%).

*HIV risk behaviours*: No prevalence data regarding drug use in Indian prisons were identified. Two sources identified denial of the possibility of drug use in prisons as an impediment to addressing HIV in prisons^12^,^18^.

Sex between inmates was reportedly common, at least in one prison^19^. In a study conducted in Arthur Road Jail, Mumbai, 72 per cent of a sample of 752 (75 employees and 677 inmates) said that they thought sex between men was common in prisons and 11 per cent engaged in homosexual activity in prison^20^.

No evidence was found on the prevalence of tattooing in prisons in India. There were reports from Mumbai of interpersonal violence (involving lacerations, bites and bleeding in two or more participants), which could present risks of HIV transmission^19^.

*HIV prevalence*: No recent HIV prevalence data were identified; existing data were from the mid-to-late 1990s. One national study of HIV prevalence in prisons found that 1.7 per cent of male and 9.5 per cent of female inmates were HIV positive^21^. In other studies HIV prevalence in individual prisons ranged from 0.5 to 6.9 per cent (Table). Eighteen of 27 inmates who died at Arthur Road Jail, Mumbai, in a six month period were HIV positive^29^. No information on HIV transmission in Indian prisons was found.

**Table.** Information on HIV prevalence in Indian prisons

Location	Year	Sample size (N)	HIV prevalence (%)
Nationally^22^	2000	Unknown	1.7 (total) 9.5 (females)
Amritsar Central Jail^23^	2003	500	2.4
Central Prison, Bangalore, south India^24^	1993	1114	1.8 (males)
Ghaziabad^25^	1999	249	1.3 (inmates aged 15 to 50 yr)
West Bengal^26^	2000	384	2.3
Orissa, 3 prisons^27^	1994-1995	377	6.9
Madras (now Chennai)^28^	1995	Unknown	3.5
Madurai^28^	1994 -1995	Unknown	4.3 (total) 2 (male) 14.2 (female)
Thirunelveli^28^	1995	Unknown	0.5

Superscript numerals denote reference numbers

*HIV prevention: Education*: HIV education programmes tended to be ad-hoc and relied on NGOs for facilitation. A sexual health programme, *Partnership for Sexual Health*, was conducted in 2000 in 11 jails in Andhra Pradesh. Three staff members provided HIV education, counselling, referral and medical treatment^30^. The Mumbai District AIDS Control Society and the International Labour Organisation have conducted a workplace HIV prevention programme at the Arthur Road Jail^20^. In West Bengal, the Vivekananda International Health Centre has delivered an HIV education programme in 20 prisons reaching 50,000 prisoners and staff^26^. In Gujarat, an HIV information and education programme was conducted by NGOs^31^.

*HIV prevention: Drug dependency treatment*: Many prisoners who were placed in drug treatment programmes, were on remand and, therefore, often released before completing treatment^32^. Drug offenders received at Tihar Jail, New Delhi were admitted to a de-addiction centre for detoxification and treatment of withdrawal symptoms. A psychiatrist worked with prisoners for approximately one week^33^. In 2005, there were three detoxification centres with 72 detoxification beds: 60 for adult males and 12 for adolescents.

After detoxification, drug offenders were segregated and placed in a therapeutic community run by NGOs. As many as 800 prisoners live within the therapeutic communities at Tihar^32^,^33^, where prisoners served as team leaders and supervisors. Staff members such as psychiatrists, psychologists, sociologists and social workers, served as trainers, facilitators and counsellors. Inmates were engaged in counselling, education, meditation, family therapy, anger and grief workshops and recreational activities^32^,^33^.

Tihar Jail hosts the only opioid substitution treatment programme in an Indian prison^34^. The programme commenced in 2008; no data on the number of prisoners receiving treatment were reported.

*HIV prevention: Harm reduction programmes*: A government run prison intervention in Andhra Pradesh included condom distribution^35^, but this was stopped as being against prison policy^18^. No information regarding bleach distribution was identified and there were no prison based needle and syringe programmes in India^18^,^36^.

*HIV care and treatment*: Only one source provided information about access to antiretroviral treatment (ART) while in prison. A programme providing legal aid to prisoners had assisted some HIV positive inmates to continue ART while in prison^18^.

The present findings revealed that only a few HIV prevention, care and treatment interventions have been implemented in prisons in India. Although no data were located on drug injecting in prison, internationally, injecting drug use is a transmission route for HIV inside prisons^37^–^40^. Two studies reported that sexual activity occurred in prisons. Unprotected sex is an HIV transmission route in prisons^41^,^42^ and the high levels of overcrowding and lack of access to condoms in Indian prisons may be conducive to unprotected sex. Distribution of condoms has previously been disallowed due to legislation against sex between men^43^ ; however, this law was overturned in July 2009^44^. The provision of condoms in prison has been found to be feasible and is not associated with increases in sexual behaviour^45^,^46^.

Some limited, sporadic HIV prevalence data were available, but such data become obsolete rapidly. Regular, nation-wide or State-based collection of HIV/STI prevalence data and information regarding HIV risk behaviours is recommended. This will provide a knowledge base to plan HIV prevention, treatment and care interventions in prison. Such a system could be based on reports available from such surveys conducted in other countries, for example, the Australian National Prison Entrants’ Bloodborne Virus Survey^47^.

Limited HIV prevention programmes were available in Indian prisons. HIV information, education and communication programmes such as those in prisons in Mumbai, Andhra Pradesh should be expanded to all prisons across the country. Improving access to drug dependency treatment, including scaling-up of opioid substitution treatment, would be another positive step^48^. Harm reduction strategies, including condom distribution programmes and needle and syringe programmes, will further assist in reducing the risk of HIV transmission in prison. Several developing countries have introduced methadone maintenance in prison. These countries include Indonesia, Iran and Moldova^48^.

Treatment of HIV and STI is also important in stemming HIV in prisons. Voluntary counselling and testing should be promoted and access to HIV and STI treatment improved.

UNODC recommended that the Government of India initiate a process of inquiry in major prisons in India, and where necessary, set up the required facilities for the treatment of drug users^32^. Large numbers of prisoners under trial pass through India’s prisons each year. Legal reforms to increase non-custodial options for monitoring offenders who have been charged but not yet tried are strongly recommended.
